# Tear Strength Analysis of MDX4-4210 and A-2186 Silicones with Different Intrinsic Pigments Incorporated by Mechanical and Industrial Methods

**DOI:** 10.1155/2019/2573095

**Published:** 2019-12-21

**Authors:** Marcelo Coelho Goiato, Adhara Smith Nobrega, Emily Vivianne Freitas da Silva, Daniela Micheline dos Santos, André Pinheiro de Magalhães Bertoz, Mariana Vilela Sonego, Clovis Lamartine de Moraes Melo Neto

**Affiliations:** ^1^Aracatuba Dental School, São Paulo State University (UNESP), Bucal Oncology Center, Aracatuba, Sao Paulo, Brazil; ^2^Aracatuba Dental School, São Paulo State University (UNESP), Department of Dental Materials and Prosthodontics, Aracatuba, Sao Paulo, Brazil; ^3^Aracatuba Dental School, São Paulo State University (UNESP), Department of Pediatric and Social Dentistry, Araçatuba, São Paulo, Brazil

## Abstract

**Objective:**

The aim of this study was to evaluate the tear strength of MDX4-4210 and A-2186 silicones with different intrinsic pigments incorporated by mechanical and industrial methods, comparing nonaged and aged groups.

**Materials and Methods:**

Twenty-four groups were created according to the American Society for Testing and Materials D-624/type C, half nonaged and half aged (*n* = 10): bronze mechanical MDX4-4210, bronze industrial MDX4-4210, black mechanical MDX4-4210, black industrial MDX4-4210, pink mechanical MDX4-4210, pink industrial MDX4-4210, bronze mechanical A-2186, bronze industrial A-2186, black mechanical A-2186, black industrial A-2186, pink mechanical A-2186, and pink industrial A-2186. All specimens were submitted to tear strength analysis. Data were submitted to the ANOVA and Tukey test (*p* < 0.05).

**Results:**

An increase in the tear strength values was observed only for the bronze and black MDX4-4210, comparing nonaged and aged silicones (*p* < 0.05), regardless of the manufacturing method. There was a difference in all comparisons between MDX4-4210 and A-2186 silicones with the same pigment type (*p* < 0.05), regardless of the manufacturing method. In all cases, there was no difference in the manufacturing method comparing the MDX4-4210 or A-2186 groups with the same pigment.

**Conclusion:**

Accelerated aging did not influence the tear strength in all aged A-2186 silicones and in aged pink industrial and mechanical MDX4-4210 silicones. The other MDX4-4210 groups had an increase in the results after aging. In all cases compared, the A-2186 groups had higher tear strength values than the MDX4-4210 groups. Mechanical and industrial methods can be used for silicone preparation, without changing the tear strength.

## 1. Introduction

Maxillofacial prostheses play a fundamental role in the rehabilitation of patients with deformities resulting from trauma, congenital origins, or surgical procedures [1–9]. Restoring a patient's appearance allows them to improve their self-esteem, helping them lead a normal life [4, 7, 8].

Currently, most maxillofacial prostheses are made of silicone elastomers [8, 10]. These are the most accepted materials due to the ease of handling, chemical inertia, proper strength, durability, biocompatibility [4, 11, 12], flexibility, texture similar to that of the human skin, and heat stability. Additionally, these materials repel water, blood, and organic materials, thus eliminating bacterial colonization [8].

The thin margins of silicone prostheses are usually glued to the patient's face using a medical adhesive. The thin margins of this type of prosthesis are susceptible to tearing as the prosthesis is removed from the attached facial tissue [4, 6]. In addition, maxillofacial prostheses can be retained by implants. For this, a layer of acrylic resin is adhered to the silicone to facilitate the bonding of the silicone to the implant. However, there is no good chemical adhesion between the silicone and the resin, which can result in the tearing of the silicone during the removal of the prosthesis [7].

An increase in the tear strength of a silicone can promote an increase in the esthetic quality of the facial prosthesis since it allows the use of thinner margins, with greater possibility of elongation and lesser chance of rupture [4]. According to Rai et al. and Aziz et al., the most important property for maxillofacial prostheses is the tear strength, from a clinical point of view [4, 6].

Silicone elastomers may be influenced by a variety of factors, such as intrinsic pigmentation [4], ultraviolet (UV) light [8, 10], and/or the manufacturing method (pigment incorporation method into silicone) [13]. When the pigment is mixed with silicone, bubbles may be incorporated into the material. These bubbles may influence the mechanical properties (e.g., tear strength) of a silicone [13]. The method for incorporating pigment into silicone can help minimize bubble incorporation [13]. Therefore, the study of methods of incorporating the intrinsic pigment to the silicone is very important for the durability of a facial prosthesis.

The Silastic MDX4-4210 is an elastomer widely used for facial rehabilitation [14–16]. In the studies of Dootz et al., Sanchez et al., and Haug et al., the tear strength of MDX4-4210 silicone is compared with that of A-2186 silicone [10, 15, 17]. However, these comparisons did not evaluate the incorporation of different pigments and/or different methods of manufacturing of these materials. Therefore, the aim of this study was to evaluate the tear strength of MDX4-4210 and A-2186 silicones with different intrinsic pigments incorporated by mechanical and industrial methods, comparing nonaged and aged groups.

## 2. Materials and Methods

For this study, Silastic MDX4-4210 (Dow Corning Corporation Medical Products, USA) and A-2186 (Factor II, AZ, USA) silicones were prepared with the addition of intrinsic pigments. Bronze (Functional Intrinsic II–215, Factor II, USA) and black (Black Functional Intrinsic II–205, Factor II, USA) pigments specific for characterization of prostheses were used. In addition, a new pink pigment (Orbital Colors, Brazil) was tested. The pink pigment was formed by the union of yellow, red, and black pigments and white opacifier. All tested pigments had an organic origin and the white opacifier (TiO_2_) had a mineral origin.

The silicones and pigments were weighed on a digital analytical balance (Adventurer, Ohaus Corporation, USA). Each pigment from Factor II (bronze and black) corresponded to 0.2% of the weight of its respective silicone [2]. For the pink pigment, the pigments that constituted it corresponded to 0.6% (white) [18], 0.122% (yellow), 0.03% (red), and 0.006% (black) [2] of the silicone weight.

**Figure 1:**
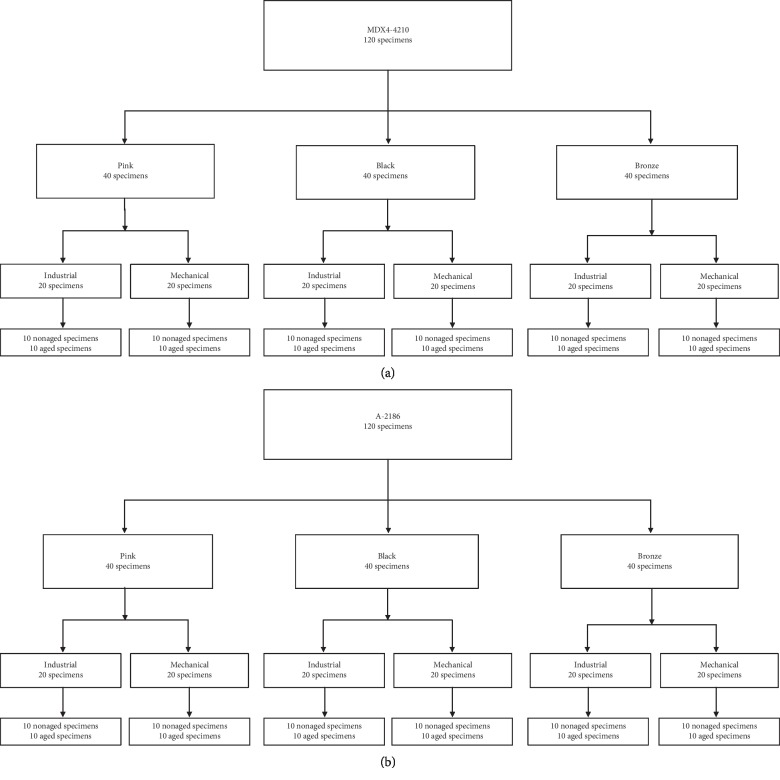
(a) Flowchart of MDX4-4210 specimens. (b) Flowchart of A-2186 specimens.

**Figure 2:**
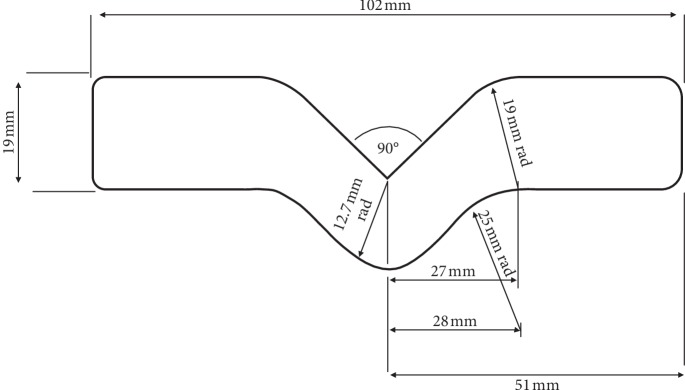
Dimensions of specimens based on ASTM D-624 (type C).

A total of 240 specimens were manufactured (Figures 1(a) and 1(b)). The silicones were manipulated according to each manufacturer's instructions at a temperature of 23 ± 2°C [2, 8, 9, 12]. Half of the specimens were fabricated by the mechanical method of incorporating the intrinsic pigment to the silicone. For this, the pigment was manually mixed with the silicone for 15 seconds, followed by a vacuum spatulation at 425 rpm in a mechanical spreader (Polidental Ind. e Com. Ltda, Brazil) until the mass became homogeneous. Subsequently, the silicone was inserted into a metal matrix. The matrix was closed and submitted to 1 ton for 10 minutes. After this period, the silicone contained in the matrix was placed on a bench and exposed to the environment (29°C) for 72 hours, until the complete polymerization of the material. The other half of the specimens was fabricated at Orbital Colors using the industrial method of incorporating the intrinsic pigment to the silicone by means of a grinding machine (CHSG/3-Roll Mill, Chemieland, China). The pigment was mixed with the silicone in the machine. Then, the silicone was inserted into the matrix, following the same procedure as the previous method. In this method, the *Deutsches Institut für Normung* (DIN—53235) was used. Specimens were made in matrices with standard dimensions and had a 2 mm thickness (Figure 2).

Twenty-four groups were created, half nonaged and half aged (*n* = 10): bronze mechanical MDX4-4210, bronze industrial MDX4-4210, black mechanical MDX4-4210, black industrial MDX4-4210, pink mechanical MDX4-4210, pink industrial MDX4-4210, bronze mechanical A-2186, bronze industrial A-2186, black mechanical A-2186, black industrial A-2186, pink mechanical A-2186, and pink industrial A-2186.

The specimens were submitted to the accelerated aging test using an accelerated aging chamber (Equilam, Brazil) according to the American Society for Testing and Materials—Designation G53-96) [19]. The lamps (UVB 313, 40 Watts, Equilam, Brazil) emitted UVB light at a wavelength of 313 nm and irradiation of 0.49 W/m^2^/nm. Then, they were subjected to alternating periods of UVB light and condensation using oxygen-saturated distilled water, under conditions of heat and 100% humidity. Each aging cycle lasted 12 hours. In the first 8 hours, the temperature was maintained at 60 ± 3°C and the UV light was imputed onto the specimens. In the last 4 hours, the temperature was maintained at 45 ± 3°C and a condensation period occurred without light [2, 3, 8, 11]. The aging was performed for a total of 1008 hours, and the deterioration caused by rain, dew, and UV light from the sun was simulated [2, 3, 8, 11]. This period corresponded to approximately one year of prosthesis use [11]. The specimens that would not be aged were stored in a dark chamber at room temperature (23 ± 2°C) and 50 ± 5% relative humidity for 1008 hours [20].

**Figure 3:**
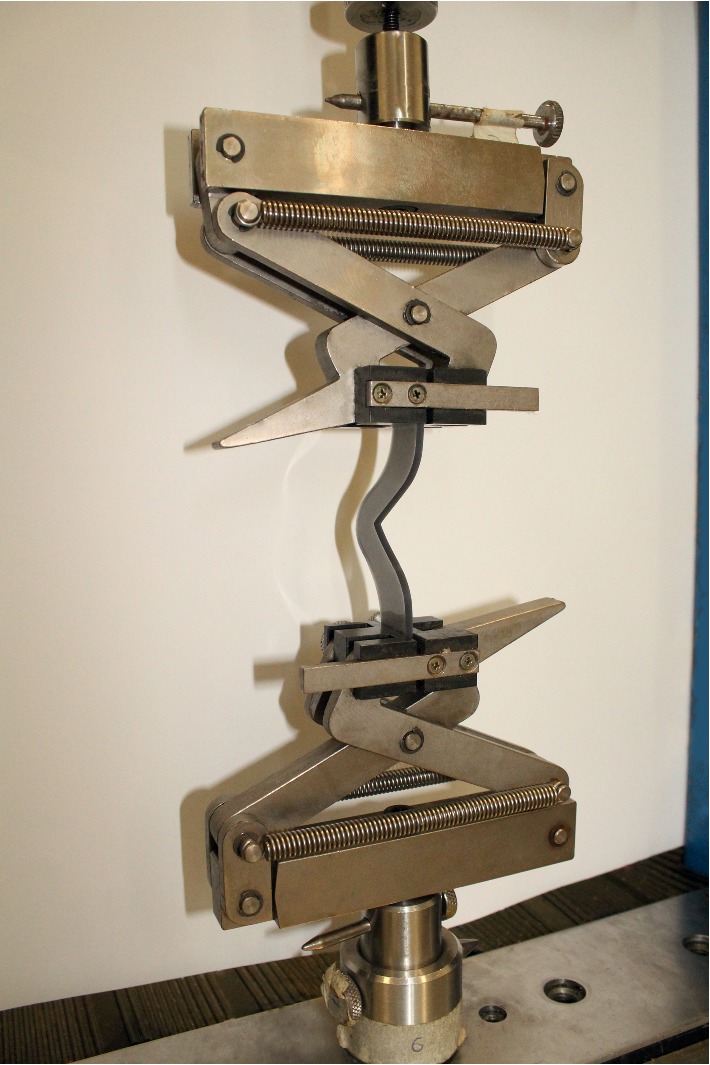
Specimen positioned for tear test.

All specimens were tested using a universal testing machine (EMIC, Instron, Brazil) (Figure 3). Specimens were stretched at a rate of 500 mm/min. The maximum tear strength value was recorded in Newtons (N). The process was determined according to the American Society for Testing and Materials (ASTM) D-624 (type C) [4, 14, 17]. The formula *T* = *F*/*D* was used, with *F* being the maximum force required to break the specimen and *D* being the thickness of the specimen. The results were obtained in N/mm.

All data were analyzed using the Statistical Package for Social Sciences 20.0 (SPSS-IBM Corp., USA). The normal distribution was verified through the Shapiro–Wilk test. Data were analyzed through the analysis of variance (ANOVA) and the Tukey test, with a level of significance of 5%.

## 3. Results

Tables 1–5 show the mean and standard deviation (SD) of each group. In Table 1, the tear strength of nonaged and aged silicone groups with the same pigmentation was compared. These comparisons were made within each manufacturing method. An increase in the tear strength values was observed only for the bronze and black MDX4-4210 silicone, for both manufacturing methods (*p* < 0.05).

Table 2 shows all nonaged groups, comparing MDX4-4210 and A-2186 silicones, based on the same pigment type. These comparisons were made within each manufacturing method. The tear strength values were higher for the A-2186, compared with the MDX4-4210 silicone (*p* < 0.05).

Table 3 shows all aged groups, comparing MDX4-4210 and A-2186 silicones, based on the same pigment type. These comparisons were made within each manufacturing method. In all cases, the tear strength values were higher for the A-2186, compared with the MDX4-4210 silicone (*p* < 0.05).

Table 4 shows all non-aged groups, comparing the mechanical and industrial methods, within the MDX4-4210 and A-2186 groups, with the same pigment type. There was no difference between the manufacturing methods (*p* > 0.05).

Table 5 shows all aged groups, comparing the mechanical and industrial methods, within the MDX4-4210 and A-2186 groups, with the same pigment type. There was no difference between the manufacturing methods (*p* > 0.05).

## 4. Discussion

In this study, it was possible to observe a statistically significant increase in the tear strength of aged bronze and black MDX4-4210 silicone, for both manufacturing methods, when compared with the respective nonaged groups. These results show that there was an increase in the tear strength of this silicone, regardless of the pigment color (lighter or darker). Presumably, the polymerization of this material was incomplete after its manufacture, and the UV light continued this process [8]. Despite this, there was no increase in this property for aged pink industrial and mechanical MDX4-4210 groups (Table 1). This could have occurred because the new pink pigment has TiO_2_ in its constitution. This component has a high refractive index and is used in the manufacture of sunscreens to protect human skin against UV rays [8, 11, 18]. Therefore, the TiO_2_ must have prevented UV rays from influencing the polymerization of pink industrial and mechanical MDX4-4210 groups.

For the A-2186 groups, regardless of the pigment or manufacturing method, there was no significant statistical difference in tear strength comparing nonaged and aged groups (Table 1). This suggests that the polymerization of this silicone has been complete since its manufacture. Therefore, the UV rays had an insignificant influence on the same.

Regardless of the manufacturing method, the A-2186 silicone showed significantly higher tear strength values compared with the MDX4-4210 silicone, when the same pigment was used (Tables 2 and 3). This difference could have occurred due to the higher filler loading and/or higher molecular weight of the dimethylsiloxane polymer from the A-2186 silicone [16]. Despite using different methodologies, this result corroborates the studies performed by Dootz et al. [10], Sanchez et al. [15], and Haug et al. [17]. According to Sanchez et al., higher values of tear strength of the A-2186 silicone compared with the MDX4-4210 silicone may clinically indicate higher prosthesis longevity [15].

When comparing mechanical and industrial methods for each silicone with the same pigment, there was no statistically significant difference (Tables 4 and 5). This may have occurred because these methods generated a similar homogeneous mixture between the silicone and pigment, with minimal and similar incorporation of bubbles during silicone handling. It is important to emphasize that the industrial method required a grinding machine and DIN standardization. In addition, the grinding machine and DIN standardization required trained professionals. These factors increase the final cost and production time of the prosthesis for the patient. Therefore, the use of the mechanical method can be more economically advantageous and faster.

This study had the limitation of the evaluation of only one property (tear strength) in the facial silicones. Therefore, there is a need for other studies evaluating other properties.

## 5. Conclusion

Accelerated aging did not influence the tear strength in all aged A-2186 silicones and in aged pink industrial and mechanical MDX4-4210 silicones. The other MDX4-4210 groups had an increase in the results after aging. In all cases compared, the A-2186 groups had higher tear strength values than the MDX4-4210 groups. Mechanical and industrial methods can be used for silicone preparation, without changing the tear strength.

## Figures and Tables

**Table 1:** Mean values ± standard deviation (SD) (N/mm) of tear strength values comparing nonaged and aged silicones.

Manufacturing method	Pigment/Silicone	Nonaged groups	Aged groups	*p* value
		Mean ± SD	Mean ± SD	
Mechanical	Bronze A-2186	68.89 ± 7.74	72.12 ± 11.17	0.387
	Bronze MDX4-4210	27.44 ± 2.45	41.16 ± 4.31	0.001^*∗*^
	Black A-2186	63.79 ± 14.21	64.87 ± 13.91	0.773
	Black MDX4-4210	29.40 ± 2.54	41.25 ± 3.82	0.002^*∗*^
	Pink A-2186	63.11 ± 12.74	65.56 ± 12.83	0.513
	Pink MDX4-4210	28.02 ± 7.64	31.65 ± 2.05	0.340

Industrial	Bronze A-2186	66.24 ± 9.99	73.10 ± 11.56	0.067
	Bronze MDX4-4210	26.46 ± 1.86	37.82 ± 3.52	0.003^*∗*^
	Black A-2186	67.81 ± 7.84	65.17 ± 11.85	0.484
	Black MDX4-4210	29.49 ± 3.23	42.53 ± 6.66	0.001^*∗*^
	Pink A-2186	69.18 ± 11.17	70.46 ± 6.07	0.722
	Pink MDX4-4210	27.04 ± 1.07	31.06 ± 4.11	0.282

^*∗*^Statistically significant difference (*p* < 0.05, Tukey).

**Table 2:** Mean values ± standard deviation (SD) (N/mm) of tear strength values comparing nonaged MDX4-4210 and A-2186 silicones, based on same pigmentation.

Manufacturing method	Pigment	Silicone	Nonaged groups	*p* value
			Mean ± SD	
Mechanical	Bronze	MDX4-4210	27.44 ± 2.45	<0.001^*∗*^
		A-2186	68.89 ± 7.74	
	Black	MDX4-4210	29.40 ± 2.54	<0.001^*∗*^
		A-2186	63.79 ± 14.21	
	Pink	MDX4-4210	28.02 ± 7.64	<0.001^*∗*^
		A-2186	63.11 ± 12.74	

Industrial	Bronze	MDX4-4210	26.46 ± 1.86	<0.001^*∗*^
		A-2186	66.24 ± 9.99	
	Black	MDX4-4210	29.49 ± 3.23	<0.001^*∗*^
		A-2186	67.81 ± 7.84	
	Pink	MDX4-4210	27.04 ± 1.07	<0.001^*∗*^
		A-2186	69.18 ± 11.17	

^*∗*^Statistically significant difference (*p* < 0.05, Tukey).

**Table 3:** Mean values ± standard deviation (SD) (N/mm) of tear strength values comparing aged MDX4-4210 and A-2186 silicones, based on same pigmentation.

Manufacturing method	Pigment	Silicone	Aged groups	*p* value
			Mean ± SD	
Mechanical	Bronze	MDX4-4210	41.16 ± 4.31	<0.001^*∗*^
		A-2186	72.12 ± 11.17	
	Black	MDX4-4210	41.25 ± 3.82	<0.001^*∗*^
		A-2186	64.87 ± 13.91	
	Pink	MDX4-4210	31.65 ± 2.05	<0.001^*∗*^
		A-2186	65.56 ± 12.83	

Industrial	Bronze	MDX4-4210	37.82 ± 3.52	<0.001^*∗*^
		A-2186	73.10 ± 11.56	
	Black	MDX4-4210	42.53 ± 6.66	<0.001^*∗*^
		A-2186	65.17 ± 11.85	
	Pink	MDX4-4210	31.06 ± 4.11	<0.001^*∗*^
		A-2186	70.46 ± 6.07	

^*∗*^Statistically significant difference (*p* < 0.05, Tukey).

**Table 4:** Mean values ± standard deviation (SD) (N/mm) of tear strength values comparing mechanical and industrial methods of incorporation of pigments to nonaged MDX4-4210 and A-2186 silicones, based on same pigmentation.

Silicone	Pigment	Manufacturing method	Nonaged groups	*p* value
			Mean ± SD	
MDX4-4210	Bronze	Mechanical	27.44 ± 2.45	0.621
		Industrial	26.46 ± 1.86	
	Black	Mechanical	29.40 ± 2.54	0.975
		Industrial	29.49 ± 3.23	
	Pink	Mechanical	28.02 ± 7.64	0.784
		Industrial	27.04 ± 1.07	

A-2186	Bronze	Mechanical	68.89 ± 7.74	0.481
		Industrial	66.24 ± 9.99	
	Black	Mechanical	63.79 ± 14.21	0.284
		Industrial	67.81 ± 7.84	
	Pink	Mechanical	63.11 ± 12.74	0.105
		Industrial	69.18 ± 11.17	

^*∗*^Statistically significant difference (*p* < 0.05, Tukey).

**Table 5:** Mean values ± standard deviation (SD) (N/mm) of tear strength values comparing mechanical and industrial methods of incorporation of pigments to aged MDX4-4210 and A-2186 silicones, based on same pigmentation.

Silicone	Pigment	Manufacturing method	Aged groups	*p* value
			Mean ± SD	
MDX4-4210	Bronze	Mechanical	41.16 ± 4.31	0.364
		Industrial	37.82 ± 3.52	
	Black	Mechanical	41.25 ± 3.82	0.739
		Industrial	42.53 ± 6.66	
	Pink	Mechanical	31.65 ± 2.05	0.878
		Industrial	31.06 ± 4.11	

A-2186	Bronze	Mechanical	72.12 ± 11.17	0.789
		Industrial	73.10 ± 11.56	
	Black	Mechanical	64.87 ± 13.91	0.932
		Industrial	65.17 ± 11.85	
	Pink	Mechanical	65.56 ± 12.83	0.185
		Industrial	70.46 ± 6.07	

^*∗*^Statistically significant difference (*p* < 0.05, Tukey).

## Data Availability

The data used to support the findings of this study are included within the article.

## References

[1] Goiato M. C., Pesqueira A. A., dos Santos D. M., Antenucci R. M., Ribeiro P. P. (2008). Evaluation of dimensional change and detail reproduction in silicones for facial prostheses. *Acta Odontológica Latinoamericana*.

[2] dos Santos D. M., Goiato M. C., Moreno A., Pesqueira A. A., Haddad M. F. (2011). Influence of pigments and opacifiers on color stability of an artificially aged facial silicone. *Journal of Prosthodontics*.

[3] Babu A. S., Manju V., Gopal V. K. (2018). Effect of chemical disinfectants and accelerated aging on maxillofacial silicone elastomers: an in vitro study. *Indian Journal of Dental Research*.

[4] Rai S. Y., Guttal S. S. (2013). Effect of intrinsic pigmentation on the tear strength and water sorption of two commercially available silicone elastomers. *The Journal of Indian Prosthodontic Society*.

[5] Abdelnnabi M. M., Moore D. J., Sakumura J. S. (1984). In vitro comparison study of MDX-4-4210 and polydimethyl siloxane silicone materials. *The Journal of Prosthetic Dentistry*.

[6] Aziz T., Waters M., Jagger R. (2003). Analysis of the properties of silicone rubber maxillofacial prosthetic materials. *Journal of Dentistry*.

[7] Bonatto L. D. R., Goiato M. C., da Silva E. V. F. (2017). Biocompatibility of primers and an adhesive used for implant-retained maxillofacial prostheses: an in vitro analysis. *The Journal of Prosthetic Dentistry*.

[8] Nobrega A. S., Andreotti A. M., Moreno A., Sinhoreti M. A. C., Dos Santos D. M., Goiato M. C. (2016). Influence of adding nanoparticles on the hardness, tear strength, and permanent deformation of facial silicone subjected to accelerated aging. *The Journal of Prosthetic Dentistry*.

[9] Guiotti A. M., Goiato M. C., dos Santos D. M. (2010). Evaluation of the shore a hardness of silicone for facial prosthesis as to the effect of storage period and chemical disinfection. *Journal of Craniofacial Surgery*.

[10] Dootz E. R., Koran A., Craig R. G. (1994). Physical properties of three maxillofacial materials as a function of accelerated aging. *The Journal of Prosthetic Dentistry*.

[11] Goiato M. C., Haddad M. F., Pesqueira A. A., Moreno A., Dos Santos D. M., Bannwart L. C. (2011). Effect of chemical disinfection and accelerated aging on color stability of maxillofacial silicone with opacifiers. *Journal of Prosthodontics*.

[12] Pesqueira A. A., Goiato M. C., dos Santos D. M. (2011). Effect of disinfection and accelerated aging on color stability of colorless and pigmented facial silicone. *Journal of Prosthodontics*.

[13] Hatamleh M. M., Watts D. C. (2011). Porosity and color of maxillofacial silicone elastomer. *Journal of Prosthodontics*.

[14] Santawisuk W., Kanchanavasita W., Sirisinha C., Harnirattisai C. (2013). Mechanical properties of experimental silicone soft lining materials. *Dental Materials Journal*.

[15] Sanchez R. A., Moore D. J., Cruz D. L., Chappell R. (1992). Comparison of the physical properties of two types of polydimethyl siloxane for fabrication of facial prostheses. *The Journal of Prosthetic Dentistry*.

[16] Lai J. H., Hodges J. S. (1999). Effects of processing parameters on physical properties of the silicone maxillofacial prosthetic materials. *Dental Materials*.

[17] Haug S. P., Andres C. J., Munoz C. A., Okamura M. (1992). Effects of environmental factors on maxillofacial elastomers: part III-physical properties. *The Journal of Prosthetic Dentistry*.

[18] Han Y., Kiat-amnuay S., Powers J. M., Zhao Y. (2008). Effect of nano-oxide concentration on the mechanical properties of a maxillofacial silicone elastomer. *The Journal of Prosthetic Dentistry*.

[19] ASTM (1996). *ASTM G53-96, Practice for Operating Light- and Water-Exposure Apparatus (Fluorescent UV-Condensation Type) for Exposure of Nonmetallic Materials (Withdrawn 2000)*.

[20] Hatamleh M. M., Polyzois G. L., Silikas N., Watts D. C. (2011). Effect of extraoral aging conditions on mechanical properties of maxillofacial silicone elastomer. *Journal of Prosthodontics*.

